# The Associations between Serum Brain-Derived Neurotrophic Factor, Potential Confounders, and Cognitive Decline: A Longitudinal Study

**DOI:** 10.1371/journal.pone.0091339

**Published:** 2014-03-26

**Authors:** Jasmine Nettiksimmons, Eleanor M. Simonsick, Tamara Harris, Suzanne Satterfield, Caterina Rosano, Kristine Yaffe, for the Health ABC Study

**Affiliations:** 1 Department of Psychiatry, University of California San Francisco, San Francisco, California, United States of America; 2 Clinical Research Branch, National Institute on Aging, Baltimore, Maryland, United States of America; 3 Laboratory of Epidemiology, Demography, and Biometry, Intramural Research Program, National Institute on Aging, Bethesda, Maryland, United States of America; 4 Department of Preventive Medicine, University of Tennessee Health Science Center, Memphis, Tennessee, United States of America; 5 Center for Aging and Population Health, Department of Epidemiology, University of Pittsburgh, Pittsburgh, Pennsylvania, United States of America; 6 Departments of Psychiatry, Neurology, and Epidemiology and Biostatistics, University of California San Francisco, San Francisco Veterans Affairs Medical Center, San Francisco, California, United States of America; Rutgers University, United States of America

## Abstract

Brain-derived neurotrophic factor (BDNF) plays a role in the maintenance and function of neurons. Although persons with Alzheimer’s disease have lower cortical levels of BDNF, evidence regarding the association between circulating BDNF and cognitive function is conflicting. We sought to determine the correlates of BDNF level and whether BDNF level was prospectively associated with cognitive decline in healthy older adults. We measured serum BDNF near baseline in 912 individuals. Cognitive status was assessed repeatedly with the modified Mini-Mental Status Examination and the Digit Symbol Substitution test over the next 10 years. We evaluated the association between BDNF and cognitive decline with longitudinal models. We also assessed the association between BDNF level and demographics, comorbidities and health behaviors. We found an association between serum BDNF and several characteristics that are also associated with dementia (race and depression), suggesting that future studies should control for these potential confounders. We did not find evidence of a longitudinal association between serum BDNF and subsequent cognitive test trajectories in older adults, although we did identify a potential trend toward a cross-sectional association. Our results suggest that serum BDNF may have limited utility as a biomarker of prospective cognitive decline.

## Introduction

Brain-derived neurotrophic factor (BDNF) is one of a family of neurotrophins which play an important role in the development, maintenance and function of neurons [1]–[4]. In humans, BDNF levels are lower in AD patients than controls in the hippocampus and cortical regions associated with AD; in the healthy adult mouse, BDNF levels are high in brain regions associated with Alzheimer’s disease (AD) [5]–[9]. BDNF also circulates in the blood, where it appears to be stored in platelets and is released through physical or chemical platelet activation [10]. While BDNF can cross the blood-brain barrier, the extent to which blood derived BDNF levels are a reasonable proxy for BDNF in the central nervous system is unclear [11], [12].

The research community hoped that circulating BDNF could provide a less invasive, inexpensive biomarker for dementia; but so far, there is conflicting evidence in the literature regarding the association between BDNF and cognition decline and dementia. Some studies indicate serum BDNF is higher in controls than persons with mild cognitive impairment (MCI), AD or dementia [13]–[15], others indicate the opposite direction of association [16], and some studies show no association [17]–[19]. Confounding may explain some of these discrepancies – many studies examining associations between BDNF and cognitive status used basic tests of group differences with minimal controls for confounding and few have examined the longitudinal association between BDNF and cognitive change. To our knowledge, only two other studies have examined cognitive change and blood based BDNF; one study in AD patients found lower BDNF predicted more rapid cognitive decline but the other study found no association in a group of healthy older adults [20], [21]. Additional studies with careful controls for potential confounders are necessary.

In this analysis, our goal was two-fold. First, we aimed to elucidate correlates of BDNF, as they show some inconsistencies in the literature. Substantial evidence supports an association between depression and lower serum BDNF but evidence regarding correlations between BDNF and basic characteristics like sex, age, and BMI is equivocal. Second, we aimed to evaluate the association between BDNF and cognitive decline, especially after consideration of potential confounders using a large prospective cohort of cognitively healthy older adults with cognitive performance evaluated at multiple time points over a lengthy follow-up. The cohort is biracial (black and white) which allows examination of possible differences in BDNF by race, which other studies have lacked sufficient diversity to address.

## Methods

### Ethics Statement

The Institutional Review Boards at all clinical sites (University of Pittsburgh, University of Tennessee- Memphis) and the University of California – San Francisco coordinating center approved the study protocol and all participants signed informed consent.

### Participants

Participants were enrolled in the Health, Aging and Body Composition (Health ABC) study, a prospective cohort of 3075 community-dwelling black and white older adults living in Memphis, Tennessee and Pittsburgh, Pennsylvania. Participants were recruited from a random sample of Medicare-eligible adults living within designated zip codes. Participants were eligible if they reported no difficulties performing activities of daily living, walking a quarter mile, climbing 10 steps without resting, were free from life-threatening cancers, and planned to remain in the study area for at least 3 years. All patients signed an informed consent form that was approved by the institutional review boards of each clinical site and the data coordinating center. Serum BDNF levels were measured in a subset of 1000 participants, selected by stratified random sample, excluding those with possible dementia (3MS <80 at baseline). The BDNF measurements for 77 participants were missing for technical reasons, and 11 participants were missing cognitive testing at included time points leaving an analytical sample of 912.

### Measures

Serum BDNF was measured at the first follow-up visit (year 2). Fasting blood samples were obtained by standard venipuncture with serum was stored at −70°C and shipped directly to the analytical laboratory in Minneapolis, MN. Assays were performed by R&D Systems’ Analytical Testing Service, employing an enzyme-linked immunosorbent assay method. The detection limit for this assay is 1250 pg/mL. The mean inter-assay coefficient of variation is 9.2% and the mean coefficient of variation within assay is 6.5%. Covariates examined were assessed at year 2 where available. If unavailable, assessments from an adjacent year were used (e.g., platelet count was measured in year 3, drinking/smoking behavior measured at baseline). Participants were considered depressed if they scored greater or equal to 16 on the Center for Epidemiologic Studies Depression Scale (CES-D) at baseline or reported treatment for depression at baseline (medication verified). Potential covariates included demographic characteristics, cardiovascular comorbidities, Apolipoprotein ε4 carrier status, BDNF Val66Met Met(A) carrier status (SNP rs6265), health behaviors, and platelet count, which was previously found to be an important predictor of BDNF [22]. Seasonal fluctuation in serum BDNF has been reported; we examined December – May vs June – November differences, based on previously published results [23]. Literacy was measured with the Rapid Estimate of Adult Literacy in Medicine (REALM), an instrument for which individuals read common health-related words aloud (e.g., germs, prescription, diagnosis) [24]. Cognition was assessed using repeated measurement of the Modified Mini-Mental State Examination (3MS) (tested at years 3, 5, 8, 10, and 11) and the Digit Symbol Substitution Test (DSST) (tested at years 5, 8, 10, and 11) [25], [26]. The 3MS test evaluates global cognitive function and ranges from 0 to 100; the DSST mainly evaluates executive function and has a range of 0–90.

### Statistical Analysis

After examining the distribution of the BDNF measurements and finding a limited number of extreme values, we trimmed the data to exclude the top and bottom 1% to reduce the possibility of influential observations outside of the typical range unduly impacting the model results. This excluded ten values at or above 47.3 ng/mL (range: 47.3–63.1) and ten values at or below 5.5 ng/mL (range: 2.3–5.5), resulting in a final data range of 5.6 ng/mL –47.25 ng/mL. The association between BDNF and participant demographic and comorbid characteristics was characterized using BDNF tertiles and tested with Chi-square tests and ANOVA, where appropriate. We constructed a multivariate linear regression model using covariates with p<0.20 to examine the association between predictors and BDNF after simultaneous adjustment.

We assessed the relationship between BDNF and cognitive test scores using linear mixed-effects regression models with random slopes and intercepts. BDNF and platelet count were standardized so that coefficients could be interpreted as change in cognition associated with a one standard deviation increase in BDNF or platelet count. We examined the relationship in both unadjusted and adjusted models. The adjusted model contained covariates which differed significantly between BDNF tertiles (p≤0.05), covariates which were significant predictors of BDNF in the adjusted model (p≤0.05), and other covariates known to be important in modeling cognitive scores (study site, age, APOE ε4, race, literacy, and education).

## Results

### Correlates of BDNF

BDNF measurements were generally normally distributed with a small number of extreme values in the right tail. As such, the data was trimmed to exclude the top and bottom 1% of BDNF observations. BDNF tertiles were significantly associated with platelet count, sex, race, Met carrier (Val66Met genotype), history of MI, and smoking (Table 1). Females, blacks, smokers, and those with high platelet counts had higher BDNF. History of myocardial infarction (MI) and carrying the rarer Met allele was associated with lower BDNF. Although depression did not quite meet the p<0.20 cut-off from Table 1 (p = 0.22), we included it in the multivariate model predicting BDNF because of its strong association with BDNF in the literature. We found that race, depression, and platelet count were significant predictors of BDNF after simultaneous adjustment in a multivariate model. BDNF levels in blacks were 1.81 ng/mL higher than whites (95% CI: 1.19, 2.43) and were 2.84 ng/mL lower in participants with depression (95% CI:−5.17, −0.51). A one standard deviation increase in platelet count was associated with a 1.81 ng/mL increase in BDNF levels (95% CI: 1.19, 2.43). The BDNF estimate for females was 1.04ng/mL higher, although it was no longer significant (95% CI:−0.17, 2.24). Smoking, history of MI, and Met allele carrier status were no longer significant after adjustment for other factors.

**Table 1:** Distribution of participant characteristics by BDNF tertile.

	BDNF Tertiles			
	Low (N = 296)	Middle (N = 297)	High (N = 299)	
BDNF (ng/mL)				
Mean (SD)	15.1 (3.6)	23.1 (1.9)	32.5 (5.0)	
Range	5.56 – 19.95	19.97 – 26.49	26.51 – 47.25	
**Continuous**	**Mean (SD)**	**Mean (SD)**	**Mean (SD)**	**p-value**
3MS (year 3)	90.2 (7.0)	89.4 (7.4)	89.4 (7.7)	0.33
DSST (year 5)	32.0 (14.7)	31.9 (14.3)	32.2 (13.8)	0.97
Age (years)	74.9 (2.9)	74.9 (2.9)	75.2 (2.9)	0.46
Platelet count (thousands/uL)	205.9 (55.9)	221.2 (54.1)	235.7 (63.8)	<0.001
**Categorical**	**% (N)**	**% (N)**	**% (N)**	**p-value**
Female	47.0 (139)	54.9 (163)	63.9 (191)	<0.001
Black	44.6 (132)	54.9 (163)	61.5 (184)	<0.001
Education (<HS)	31.8 (94)	35.5 (105)	38.7 (115)	0.21
Literacy (<9^th^ grade)	28.7 (85)	29.6 (88)	35.1 (105)	0.19
APOE ε4	30.5 (85)	30.7 (85)	30.3 (87)	1.00
Val66Met Met(A) carrier	25.2 (69)	17.8 (48)	18.9 (53)	0.07
History of MI	25.3 (75)	17.2 (51)	18.4 (55)	0.03
Hypertension	62.5 (185)	59.3 (176)	60.9 (182)	0.72
CVD	11.5 (34)	10.4 (31)	11.4 (34)	0.91
High Cholesterol	48.9 (136)	55.1 (158)	57.2 (162)	0.12
Diabetes	40.5 (120)	38.7 (115)	37.1 (111)	0.69
Depression	8.1 (24)	6.1 (18)	4.7 (14)	0.22
Obese	23.0 (68)	28.6 (85)	29.1 (87)	0.17
Current smoker	6.4 (19)	11.8 (35)	11.4 (34)	0.05
Drinking (>1 drink/day)	6.4 (19)	6.1 (18)	7.7 (23)	0.71
Walking (>150 mins/week)	30.5 (90)	28.3 (84)	24.4 (73)	0.24
Season (Winter/Spring vs Summer/Fall)	35.1 (172)	32.5 (297)	32.5 (299)	0.40

P-values come from ANOVA and Chi-square tests, as appropriate. BDNF = brain derived neurotrophic factor, 3MS = Teng Modified Mini-Mental Status Exam, DSST = Digit Symbol Substitution Test, CVD = cerebrovascular disease, MI = myocardial infarction.

### Cognitive Decline

The median number of 3MS observations per person was 4 (range: 1–5); the median number of DSST observations per person was 3 (range: 1– 4). In both unadjusted and adjusted analyses, we found no significant association between BDNF and performance on either cognitive test, cross-sectionally or longitudinally (Table 2). There was a slight trend toward higher cross-sectional performance on DSST associated with higher BDNF, but it was not significant (p = 0.09). Performing the same analysis using the log of BDNF, rather than trimmed BDNF values, resulted in the same conclusions. There were no significant interactions between race and BDNF for either cognitive test. A recent study on the association between dementia treatment and serum BDNF found that treatment increased BDNF levels in persons with AD [27]. Four participants in this sample were prescribed dementia medications throughout the follow-up period; results excluding these individuals did not differ appreciably from the primary results.

**Table 2:** Estimates of the association between BDNF and cross-sectional and longitudinal cognitive test scores (change in test score per 1 SD increase in BDNF).

	N	Cross-sectional			Longitudinal	
		Per 1 SD increase in BDNF	p-value		Per 1 SD increase in BDNF	p-value
**3MS**						
Unadjusted	892	−0.44 (−0.97, 0.09)	0.11		−0.02 (−0.10, 0.07)	0.71
Adjusted	792	0.10 (−0.41, 0.61)	0.70		−0.02 (−0.11, 0.07)	0.73
**DSST**						
Unadjusted	892	0.09 (−1.20, 1.39)	0.89		−0.07 (−0.20, 0.06)	0.31
Adjusted	792	1.08 (−0.18, 2.33)	0.09		−0.08 (−0.22, 0.06)	0.27

Unadjusted model contained: BDNF (standardized), time, BDNF*time.

Adjusted model contained: BDNF (standardized), race, sex, history of myocardial infarction, smoking, platelet count (standardized), APOE ε4, age at enrollment, education<high school, literacy <9^th^ grade, depression, time, and all covariate interactions with time.

Regression model estimates and 95% confidence intervals from linear mixed effects models are provided.

## Discussion

Serum BDNF showed strong associations with race, platelet count, and depression after adjusting for other factors, and weak associations with a number of other participant characteristics. We found that blacks had higher levels of BDNF than whites, a unique finding which has not been addressed in the literature so far. It is unclear whether the race differential in BDNF is due to unmeasured factors differing between blacks and whites or true biological differences. The strong association between BDNF and platelet count is consistent with theory that BDNF in serum is derived directly from platelets. Age did not differ significantly among serum BDNF tertiles which is in concordance with results from some studies [13]–[16], [28]–[30] and in conflict with others [19], [31], [32], although two of the studies which found an age effect included a wider age range than our study. Initially, we found that sex differed significantly between BDNF tertiles but sex was not a significant predictor of BDNF in a model adjusted for other covariates.

We did not identify any longitudinal associations between BDNF and cognition; this is in agreement with the other longitudinal study of blood based BDNF in cognitively normal older adults [21]. However, a longitudinal study using CSF-derived BDNF found that lower baseline CSF BDNF levels were significantly associated with greater annual decline on several tests of memory and category fluency in cognitively normal older adults [33]. This contrast may suggest that blood based measures of BDNF are not representative of CSF BDNF levels. A study comparing CSF and serum BDNF measurements in a sample of AD patients found that they were not correlated (r^2^ = 0.03, n = 27); however, a study comparing blood and BDNF levels in pigs and rats found significant positive correlation between the two in both pigs and rats (r^2^ = 0.41, 0.44, respectively) [13], [34]. Further study will be required to better understand the relationship between cortical, cerebrospinal fluid, and circulating levels of BDNF in humans. The trend toward a cross-sectional association and the lack of longitudinal association, combined with previous published work, may suggest that changes in BDNF occur contemporaneously with cognitive decline, as opposed to preceding decline. This would limit the utility of BDNF as a biomarker for early detection but does not preclude the potential for BDNF to play a useful diagnostic role.

Comparison of our raw and adjusted analyses demonstrates that confounders may have a profound effect on the estimate of the relationship between BDNF and cognition, particularly cross-sectionally. Many previous studies chose to use nonparametric group tests due to concerns that the BDNF distribution showed departures from normality. While extreme values and departures from normality are a concern, our results suggest that confounding may be a much greater concern, especially in the presence of an adequate sample size.

Strengths of this analysis include the prospective nature of the study, large biracial sample, and ability to control for multiple potential confounders. This analysis was limited by data collection occurring at different time points for some variables, such as platelet count. The negative impact of this is likely minimal as no covariate assessment occurred more than a year from BDNF blood draw and comparisons of platelet counts measured in years 3 and 11 suggest high intra-person correlation of platelet count over time (Spearman correlation coefficient 0.73). It is possible that our relatively healthy sample of older adults without dementia at baseline lacked sufficient variability in BDNF to detect associations reported elsewhere, although the serum BDNF range exhibited in our data is consistent with other similar studies. There is evidence that treatment for depression raises BDNF levels [35]. Our depression definition was partially based on treatment for depression, which makes it difficult to untangle the separate associations with depression and antidepressants. It is possible that our estimate of the association between BDNF and depression was attenuated due to the mixture of treated and untreated individuals. We were limited to evaluating two cognitive tests with repeated assessment in this sample (DSST and 3MS), which may not provide a complete picture of cognitive status and change.

After adjustment for confounding, our analysis provides no evidence that serum BDNF is prospectively associated with decline on DSST or 3MS. We found that serum BDNF was associated with a number of characteristics which also associated with dementia, which suggests that future studies should control for these potential confounders.

## References

[1] Alderson RF, Alterman AL, Barde YA, Lindsay RM (1990) Brain-derived neurotrophic factor increases survival and differentiated functions of rat septal cholinergic neurons in culture. Neuron 5: 297–306. 2169269 10.1016/0896-6273(90)90166-d

[2] Ghosh A, Carnahan J, Greenberg ME (1994) Requirement for BDNF in activity-dependent survival of cortical neurons. Science 263: 1618–1623. 7907431 10.1126/science.7907431

[3] Bramham CR, Messaoudi E (2005) BDNF function in adult synaptic plasticity: the synaptic consolidation hypothesis. Prog Neurobiol 76: 99–125. 16099088 10.1016/j.pneurobio.2005.06.003

[4] Fahnestock M (2011) Brain-derived neurotrophic factor: the link between amyloid-β and memory loss. Future Neurology 6: 627–639.

[5] Phillips HS, Hains JM, Armanini M, Laramee GR, Johnson SA, et al (1991) BDNF mRNA is decreased in the hippocampus of individuals with Alzheimer's disease. Neuron 7: 695–702. 1742020 10.1016/0896-6273(91)90273-3

[6] Holsinger RM, Schnarr J, Henry P, Castelo VT, Fahnestock M (2000) Quantitation of BDNF mRNA in human parietal cortex by competitive reverse transcription-polymerase chain reaction: decreased levels in Alzheimer's disease. Brain Res Mol Brain Res 76: 347–354. 10762711 10.1016/s0169-328x(00)00023-1

[7] Peng S, Wuu J, Mufson EJ, Fahnestock M (2005) Precursor form of brain-derived neurotrophic factor and mature brain-derived neurotrophic factor are decreased in the pre-clinical stages of Alzheimer's disease. J Neurochem 93: 1412–1421. 15935057 10.1111/j.1471-4159.2005.03135.x

[8] Hock C, Heese K, Hulette C, Rosenberg C, Otten U (2000) Region-specific neurotrophin imbalances in Alzheimer disease: decreased levels of brain-derived neurotrophic factor and increased levels of nerve growth factor in hippocampus and cortical areas. Arch Neurol 57: 846–851. 10867782 10.1001/archneur.57.6.846

[9] Hofer M, Pagliusi SR, Hohn A, Leibrock J, Barde YA (1990) Regional distribution of brain-derived neurotrophic factor mRNA in the adult mouse brain. EMBO J 9: 2459–2464. 2369898 10.1002/j.1460-2075.1990.tb07423.x PMC552273

[10] Fujimura H, Altar CA, Chen R, Nakamura T, Nakahashi T, et al (2002) Brain-derived neurotrophic factor is stored in human platelets and released by agonist stimulation. Thromb Haemost 87: 728–734. 12008958

[11] Pan W, Banks Wa, Fasold MB, Bluth J, Kastin aJ (1998) Transport of brain-derived neurotrophic factor across the blood-brain barrier. Neuropharmacology 37: 1553–1561. 9886678 10.1016/s0028-3908(98)00141-5

[12] Poduslo JF, Curran GL (1996) Permeability at the blood-brain and blood-nerve barriers of the neurotrophic factors NGF, CNTF, NT-3, BDNF. Molecular Brain Research 36: 280–286. 8965648 10.1016/0169-328x(95)00250-v

[13] Laske C, Stransky E, Leyhe T, Eschweiler GW, Maetzler W, et al (2007) BDNF serum and CSF concentrations in Alzheimer's disease, normal pressure hydrocephalus and healthy controls. Journal of psychiatric research 41: 387–394. 16554070 10.1016/j.jpsychires.2006.01.014

[14] Yasutake C, Kuroda K, Yanagawa T, Okamura T, Yoneda H (2006) Serum BDNF, TNF-alpha and IL-1beta levels in dementia patients: comparison between Alzheimer's disease and vascular dementia. European archives of psychiatry and clinical neuroscience 256: 402–406. 16783499 10.1007/s00406-006-0652-8

[15] Yu H, Zhang Z, Shi Y, Bai F, Xie C (2008) Association study of the decreased serum BDNF concentrations in amnestic mild cognitive impairment and the Val66Met polymorphism in Chinese Han. The Journal of clinical psychiatry: 1104–1111. 10.4088/jcp.v69n0710 18505307

[16] Angelucci F, Spalletta G, Iulio F, Ciaramella a, Salani F, et al (2010) Alzheimers Disease (AD) and Mild Cognitive Impairment (MCI) Patients are Characterized by Increased BDNF Serum Levels. Current Alzheimer Research 7: 15–20. 20205668 10.2174/156720510790274473

[17] Laske C, Stransky E, Leyhe T, Eschweiler GW, Wittorf a, et al (2006) Stage-dependent BDNF serum concentrations in Alzheimer's disease. Journal of neural transmission 113: 1217–1224. 16362629 10.1007/s00702-005-0397-y

[18] O'Bryant SE, Hobson VL, Hall JR, Barber RC, Zhang S, et al (2011) Serum brain-derived neurotrophic factor levels are specifically associated with memory performance among Alzheimer's disease cases. Dementia and geriatric cognitive disorders 31: 31–36. 21135555 10.1159/000321980 PMC3019366

[19] Ziegenhorn Aa, Schulte-Herbrüggen O, Danker-Hopfe H, Malbranc M, Hartung H-D, et al (2007) Serum neurotrophins–a study on the time course and influencing factors in a large old age sample. Neurobiology of aging 28: 1436–1445. 16879899 10.1016/j.neurobiolaging.2006.06.011

[20] Laske C, Stellos K, Hoffmann N, Stransky E, Straten G, et al (2011) Higher BDNF serum levels predict slower cognitive decline in Alzheimer's disease patients. The international journal of neuropsychopharmacology/official scientific journal of the Collegium Internationale Neuropsychopharmacologicum (CINP) 14: 399–404. 10.1017/S1461145710001008 20860877

[21] Driscoll I, Martin B, An Y, Maudsley S, Ferrucci L, et al (2012) Plasma BDNF is associated with age-related white matter atrophy but not with cognitive function in older, non-demented adults. PloS one 7: e35217. 22523577 10.1371/journal.pone.0035217 PMC3327651

[22] Bus Baa, Molendijk ML, Penninx BJWH, Buitelaar JK, Kenis G, et al (2011) Determinants of serum brain-derived neurotrophic factor. Psychoneuroendocrinology 36: 228–239. 20702043 10.1016/j.psyneuen.2010.07.013

[23] Molendijk ML, Haffmans JP, Bus BA, Spinhoven P, Penninx BW, et al (2012) Serum BDNF concentrations show strong seasonal variation and correlations with the amount of ambient sunlight. PLoS One 7: e48046. 23133609 10.1371/journal.pone.0048046 PMC3487856

[24] Murphy PW, Davis TC, Long SW, Jackson RH, Decker BC (1993) Rapid Estimate of Adult Literacy in Medicine (REALM): A quick reading test for patients. Journal of Reading 37: 124–130.

[25] Teng EL, Chui HC (1987) The Modified Mini-Mental State (3MS) examination. J Clin Psychiatry 48: 314–318. 3611032

[26] Wechsler D (1997) Wechsler Adult Intelligence Scale - Third Edition (WAIS-III). San Antonio, TX: The Psychological Corporation.

[27] Leyhe T, Stransky E, Eschweiler GW, Buchkremer G, Laske C (2008) Increase of BDNF serum concentration during donepezil treatment of patients with early Alzheimer's disease. Eur Arch Psychiatry Clin Neurosci 258: 124–128. 17990049 10.1007/s00406-007-0764-9

[28] Currie J, Ramsbottom R, Ludlow H, Nevill A, Gilder M (2009) Cardio-respiratory fitness, habitual physical activity and serum brain derived neurotrophic factor (BDNF) in men and women. Neuroscience letters 451: 152–155. 19133315 10.1016/j.neulet.2008.12.043

[29] Minelli A, Zanardini R, Bonvicini C, Sartori R, Pedrini L, et al (2011) BDNF serum levels, but not BDNF Val66Met genotype, are correlated with personality traits in healthy subjects. European archives of psychiatry and clinical neuroscience 261: 323–329. 21290143 10.1007/s00406-011-0189-3

[30] Gunstad J, Benitez A, Smith J, Glickman E, Spitznagel MB, et al (2008) Serum brain-derived neurotrophic factor is associated with cognitive function in healthy older adults. Journal of geriatric psychiatry and neurology 21: 166–170. 18503034 10.1177/0891988708316860

[31] Lang UE, Hellweg R, Gallinat J (2004) BDNF serum concentrations in healthy volunteers are associated with depression-related personality traits. Neuropsychopharmacology 29: 795–798. 14735133 10.1038/sj.npp.1300382

[32] Jung SH, Kim J, Davis JM, Blair SN, Cho H-c (2011) Association among basal serum BDNF, cardiorespiratory fitness and cardiovascular disease risk factors in untrained healthy Korean men. European journal of applied physiology 111: 303–311. 20878177 10.1007/s00421-010-1658-5

[33] Li G, Peskind ER, Millard SP, Chi P, Sokal I, et al (2009) Cerebrospinal fluid concentration of brain-derived neurotrophic factor and cognitive function in non-demented subjects. PloS one 4: e5424. 19412541 10.1371/journal.pone.0005424 PMC2671606

[34] Klein AB, Williamson R, Santini MA, Clemmensen C, Ettrup A, et al (2011) Blood BDNF concentrations reflect brain-tissue BDNF levels across species. Int J Neuropsychopharmacol 14: 347–353. 20604989 10.1017/S1461145710000738

[35] Sen S, Duman R, Sanacora G (2008) Serum brain-derived neurotrophic factor, depression, and antidepressant medications: meta-analyses and implications. Biological psychiatry 64: 527–532. 18571629 10.1016/j.biopsych.2008.05.005 PMC2597158

